# Generation of a dual-color reporter mouse line to monitor spermatogenesis *in vivo*

**DOI:** 10.3389/fcell.2014.00030

**Published:** 2014-07-23

**Authors:** Yoshinori Makino, Erina Inoue, Masashi Hada, Keisuke Aoshima, Satsuki Kitano, Hitoshi Miyachi, Yuki Okada

**Affiliations:** ^1^Laboratory of Pathology and Development, Institute of Molecular and Cellular Biosciences, The University of TokyoTokyo, Japan; ^2^Career-Path Promotion Unit for Young Life Scientists, Center for the Promotion of Interdisciplinary Education and Research, Kyoto UniversityKyoto, Japan; ^3^Department of Biotechnology, Graduate School of Agricultural and Life Sciences, The University of TokyoTokyo, Japan; ^4^Division of Molecular Pathobiology, Center for Zoonosis Control, Hokkaido UniversitySapporo, Japan; ^5^Reproductive Engineering Team, Institute for Virus Research, Kyoto UniversityKyoto, Japan; ^6^PRESTO, Japan Science and Technology AgencySaitama, Japan

**Keywords:** spermatogenesis, histones, reporter mice, transplantation, homologous, spermatogonial stem cells

## Abstract

*In vivo* fluorescent imaging technique is a strong tool to visualize the various cellular events such as the proliferation, differentiation, migration, and a lineage tracing in living cells requiring no further experimental procedure such as immunostaining. During spermatogenesis, unique and dynamic histone exchanges occur. Since the timing and types of histone exchanges defines the particular stages of spermatogenesis, visualizing certain types of histones in testes is useful not only for researching specific histone dynamics, but also for monitoring the stages of spermatogenesis *in vivo*. In this study, we report the establishment of two transgenic (Tg) mouse lines expressing histone H4-Venus (H4V) and histone H3.3-mCherry (H33C) fusion proteins in testicular germ cells, and demonstrated their utility for monitoring germ cell development *in vivo*. Because of the choice of promoter as well as the nature of these histones, H4V and H33C were exclusively expressed in the germ cells of the distinct stages, which allowed the determination of spermatogenic stages in real time. In addition, disappearance of H4V and H33C at particular stages of differentiation/fertilization also represented dynamic histone removal. Collectively, these Tg mice are a valuable resource not only for monitoring differentiation stages, but also for studying the chromatin dynamics of post-natal testicular germ cell development *in vivo*.

## Introduction

Spermatogenesis is the process of differentiation from spermatogonia to spermatozoa. Postnatal spermatogenesis originates from spermatogonial/spermatogenic stem cells (SSCs), which continuously undergo self-renewal as well as asymmetrical cell division to produce daughter cells, called spermatogonia. These cells proliferate by mitosis, and become spermatocytes, which enter meiotic stages. Post-meiotic stages are called spermiogenesis, in which haploid germ cells called spermatids dynamically change both their morphological and epigenetic properties. One of the most unique epigenetic changes during spermatogenesis is histone-protamine exchange. During spermiogenesis, most of the histones are gradually eliminated from chromatin, and replaced first by transition proteins (TNPs) and then by protamines, both of which are highly basic male germ cell-specific proteins. These steps make the spermatozoal chromain highly condensed, and are necessary for the acquisition of fertility. However, 1–20% of histones including specific histone variants are still retained in sperm nuclei. A recent study suggests transgenerational effects are caused by the retained histones as well as their modifications (Hammoud et al., 2009).

Like in other types of tissues, pulse chase experiments and live imaging techniques have been utilized for studies of spermatogenesis *in vivo*, particularly for tracing of SSCs/spermatogonial progenitor cells to monitor their lineages (Yoshida, 2012). The former is preferable for monitoring of prolonged period, as the label persists in not only the labeled cells but also in their differentiating/differentiated progenies. However, the label is irreversible, and fixation/staining procedure is sometimes required for visualization. On the other hand, the latter method is more direct to monitor the cell fate in real time. By taking advantage of these two methods, Hara et al successfully demonstrated that entire population of GFRα1-positive undifferentiated spermatogonia contains a single stem cell pool regardless their syncytial state, suggesting the stochastic fates of GFRα1-positive cells to serve the stem cells or differentiating spermatogonia (Hara et al., 2014). Another example is an application of live imaging to Sertoli cells, which are testicular somatic cells adjacent to germ cells in the seminiferous tubules. Nel-Themaat et al established two-color Tg mice, which can visualize the nuclei and plasma membrane of Sertoli cells with distinct colors. This mouse line is useful to monitor not only the cell division and migration of Sertoli cells themselves but also the association with germ cells in spatio-temporal manner (Nel-Themaat et al., 2011).

The entire spermatogenic process described above is reproducible by a transplantation of SSCs to germ cell-deficient recipient testis (Brinster and Zimmermann, 1994). The transplantation assay is useful not only for evaluating the stemness of donor cells, but also for pulse-chase studies for lineage tracing. Recently Sato et al developed *in vitro* testicular organ culture system, that enabled real-time monitoring of *in vitro* spermatogenesis as well as the successful engraftment of transplanted SSCs in *in-vitro*-cultured testes (Sato et al., 2011, 2013). For these experiments, they utilized SSCs isolated from Acr-GFP Tg mice as a reporter, which allowed them to detect the appearance of post-meiotic spermatids in real time. Meanwhile, a bioluminescence imaging, which has been widely utilized for cancer biology, is also reported to be feasible in germ cell study when it's combined with transplantation (Chen et al., 2012).

Since dynamic histone exclusion and retention occur in particular spermatogenic stages, utilizing specific histones as reporters can function as appropriate tools for monitoring spermatogenesis in real time, especially to judge the spermatogenic stages of engrafted cells by transplantation. To this end, here we report the establishment of two Tg mouse lines, which express H4V and H33C in male germ cells, and their possible application for SSC transplantation assays.

## Materials and methods

### Generation of Tg mice

Zygotes obtained from C57BL/6 mice (CLEA Japan Inc.) were used to generate Tg mice. For H4-Venus (H4V), Bacterial artificial chromosome (BAC) plasmid modification was carried out as described previously (Copeland et al., 2001). Briefly, an XhoI-NotI-5′ arm-linker-Venus-3xFlag-stop-poly A-XhoI sequence was inserted into a pL452 vector cut with SalI. Subsequently, the 3′ arm sequence was inserted into a pL451 vector with BamHI-NotI; the resultant plasmid was named pL452-H4V3xF. The loxP sequence in the RP23-296J10 BAC plasmid, which includes the H4 gene, was deleted by recombination with the EcoRI-digested fragment of the p23loxZeo plasmid. The Venus-3xFlag sequence was then inserted into the BAC plasmid at the immediate 3′ end of the H4 gene coding sequence (not containing stop codon sequences) by recombination with the NotI-digested fragment of pL452-H4V3xF. The neomycin cassette of the Venus-3xFlag-inserted BAC plasmid was deleted by Cre-recombination; the resultant BAC plasmid was named 296J10-V3F. Recombination and Cre reaction were performed in the E. coli strain EL350. After recombination, the plasmid DNA obtained was purified, and injected into the pronuclei of zygotes without linearization.

For H3.3-mCherry (H33C), a protamine 1 promoter sequence (from −465 to +89 bp, NCBI Gene ID: 19118, TSS: 10796916), an H3.3A coding sequence (NM_008210), mCherry cDNA, and a NotI cutting sequence were inserted into pPBbsr2, a Piggybac system vector with SpeI-XhoI-EcoRI-ClaI (Yusa et al., 2009), designated as pPB-Prm1 pro-H3.3-mCherry. Piggybac mRNA was prepared by *in vitro* transcription using RiboMAX-T7 kit (Promega) from pCMV-PBase, a Piggybac cDNA-encoding plasmid (Yusa et al., 2011). Circular pPB-Prm1 pro-H3.3-mCherry DNA and Piggybac mRNA were mixed at the concentration of 25 and 30 ng/μl in the injected solution, respectively, and were cytoplasmically injected to the zygotes.

The obtained pups were genotyped, and mice carrying the transgenes were heterozygously maintained by crossing with wild-type C57BL/6 mice. To obtain H4V/H33C double Tg mice, animals for H4V were crossed with those heterozygous H33C.

All experimental procedures involving animals were approved by the Animal Experiment Ethics Committees at the Graduate School of Medicine, Kyoto University (MedKyo11094) and the Institute of Molecular and Cellular Biosciences, The University of Tokyo (#23015). All experiments were conducted in accordance with the Guidelines for the Care and Use of Laboratory Animals of Kyoto University and The University of Tokyo. All efforts were made to minimize animal suffering and discomfort and to reduce the number of animals used.

### Antibodies

Antibodies used for immunostaining and western blotting were as follows; α-GENA (clone-TRA98; Bio Academia, Cat# 73-003, 1:250), α−γH2A.X (Abcam, Cat# ab22551, 1:250), α-SLA (clone-TRA54; Bio Academia, Cat# 73-001, 1:250), α-Sox9 (Millipore, Cat# AB5535, 1:250), α-DYKDDDDK (= Flag) tag (Clone No. 1E6, WAKO Japan, 1:2000), α-histone H3 (Abcam, Cat# ab1791, 1:1000), and α-histone H4 (CST, Cat# 2592S, 1:1000).

### Morphological and histological analyses of testes

Gross images of testes were obtained by a stereoscopic microscope (SZX16, Olympus) with a long-path filter for GFP (SZX2-FGFP, Olympus) and RFP (SZX2-FRFP1,Olympus). The histological images were taken using an inverted fluorescent microscope (IX-73, Olympus). For histological analysis, testes were fixed with 4% paraformaldehyde, and embedded with OCT compound (SAKURA Finetek Japan Co., Ltd) to make frozen sections. After permeabilization with 0.2% Triton X-100, primary antibodies were applied overnight at 4°C. Alexa Fluor 488 or 568 secondary antibodies (Life Technologies) were used for detection, followed by nuclear staining with Hoechst 33342 (Life Technologies). Staging of spermatogenesis was performed based on the morphological structure or the pattern of γH2A.X staining of testicular germ cells according to definitions (Russell et al., 1990; Hamer et al., 2003).

### Western blot

Nuclear extracts were prepared from equal weight of testicular pieces isolated from adult wildtype and H33C/H4V double Tg male. 20 mg of testicular tissues were first suspended with low salt buffer (10 mM Hepes-KOH, pH 7.8, 10 mM KCl, 0.1 mM EDTA, pH 8.0) containing 0.1% nonidet P-40 to eliminate cytoplasmic proteins. The pellets were then suspended with 200 μl of RIPA buffer (25 mM Tris-HCl pH7.6, 150 mM NaCl, 1% NP-40, 1% sodium deoxycholate, 0.1% SDS) followed by brief sonications to ensure the release of chromatin proteins. After centrifuge, the supernatants were subjected to 15% SDS-PAGE. After transferring to the PVDF membrane, the signals were detected by using IRDye secondary antibodies and ODYSSEY imaging system (LICOR).

### Flow cytometry

Single-cell-suspensions of whole testes were prepared from adult H4V/H33C double Tg mice by trypsin-DNase I treatment. Cells were stained with DRAQ5 (Cell Signaling Technology) to distinguish post-meiotic germ cells (1n) based on their DNA content. A BD FACS Aria II cell sorter was used to detect the presence of Venus, mCherry, and DRAQ5 simultaneously. Only cells expressing either H4V or H33C were used for negative controls and fluorescent compensation.

### Isolation, culture, and transplantation of SSCs

Isolation and *in vitro* culture of SSCs from P9 pups were performed as recently described by Aoshima et al. (2013). Cells were subject to transplantation assays at 3–4 weeks after isolation. Approximately 4 week-old WBB6F1/Kit-KitW/KitW-v/Slc male mice (Japan SLC, Inc.) were used as recipients of transplantation, as they lacked endogenous germ cells. 1–2 × 10^5^ SSCs were transplanted per testis, and the recipients were analyzed every 2 weeks to evaluate the efficiency of engraftment.

### Intra-cytoplasmic sperm injection (ICSI)

Epididymal sperm were isolated from adult H33C mice. Metaphase II (MII) oocytes were obtained from wild type C57BL/6 female mice by hyperovulation. Tail-removed sperm heads were injected into the cytoplasm of MII oocytes using a micromanipulation system (Narishige Japan) combined with a Piezo micro manipulator PMM-150HJ (PRIMETECH, Ltd.) and a fluorescent system (IX-73, Olympus). Fertilized oocytes injected with H33C sperm were monitored using the same fluorescent microscope.

## Results

### Generation of transgenic (Tg) mouse lines

**Figure 1:**
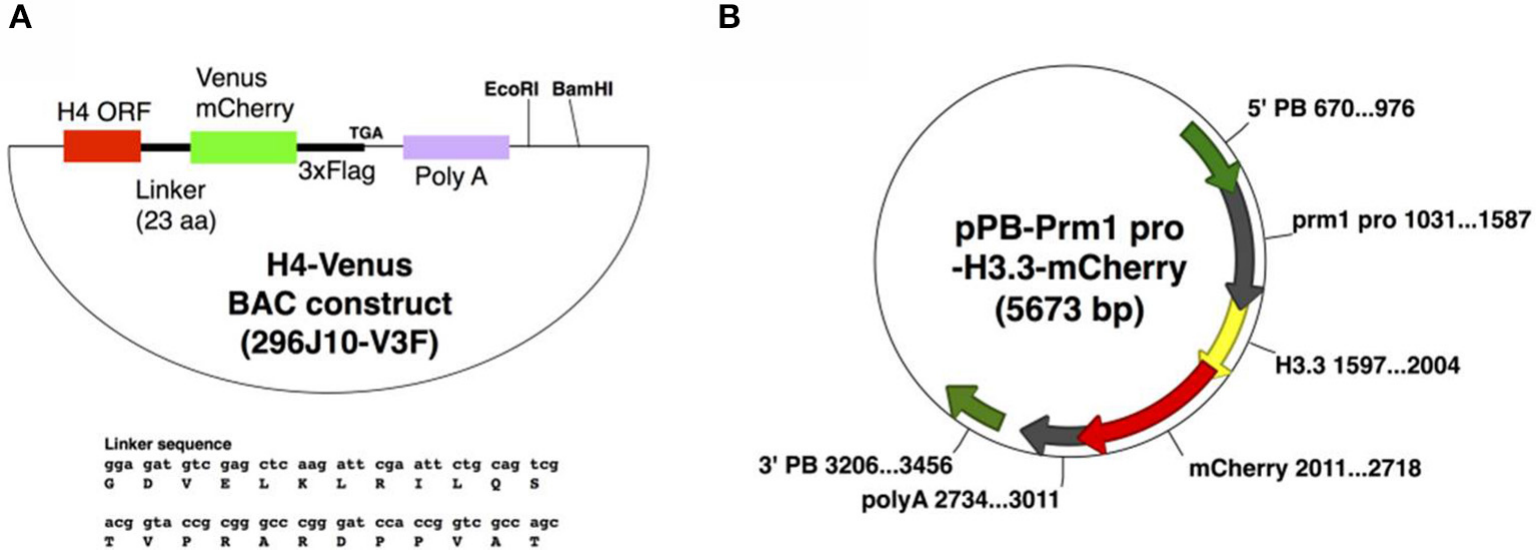
**Plasmid maps to generate H4V (A) and H33C (B) Tg mice**.

To generate H4V transgenic mice, we utilized a BAC system to maintain the endogenous expression pattern and level of the H4 histone, as ectopic and excess expression of transgene might be toxic to cells. To this end, we used BAC clone RP23-296J10 containing an entire promoter region of H4, and an open reading frame of H4 fused with Venus fluorescence-3xFlag tags as shown in Figure 1A, designated as 296J10-V3F. The DNA was injected to the pronuclei of zygotes, and two independent Tg lines were obtained. Interestingly, one of the Tg lines exhibited significantly higher expression of the H4V transgene in germ cells compared with other types of cells, although it was designed to be expressed by the endogenous H4 promoter and thus systemic ubiquitous expression was expected (data not shown). For the expression of the H33C transgene, we chose a Prm1 promoter, as H3.3 is known to be incorporated in chromatin of post-meiotic germ cells. The DNA fragment encoding a Prm-H3.3-mCherry sequence was subcloned into the Piggybac system vector pBbsr2 (Figure 1B), which enabled us to generate a transgenic line in a transposase-dependent manner (Sumiyama et al., 2010; Yusa et al., 2011). As a result, four independent Tg lines were obtained, and all of them exhibited similar properties (data not shown). Thus, line#2 was used for further analysis.

### Expression pattern of H4V and H33C in transgenic mouse testes

**Figure 2:**
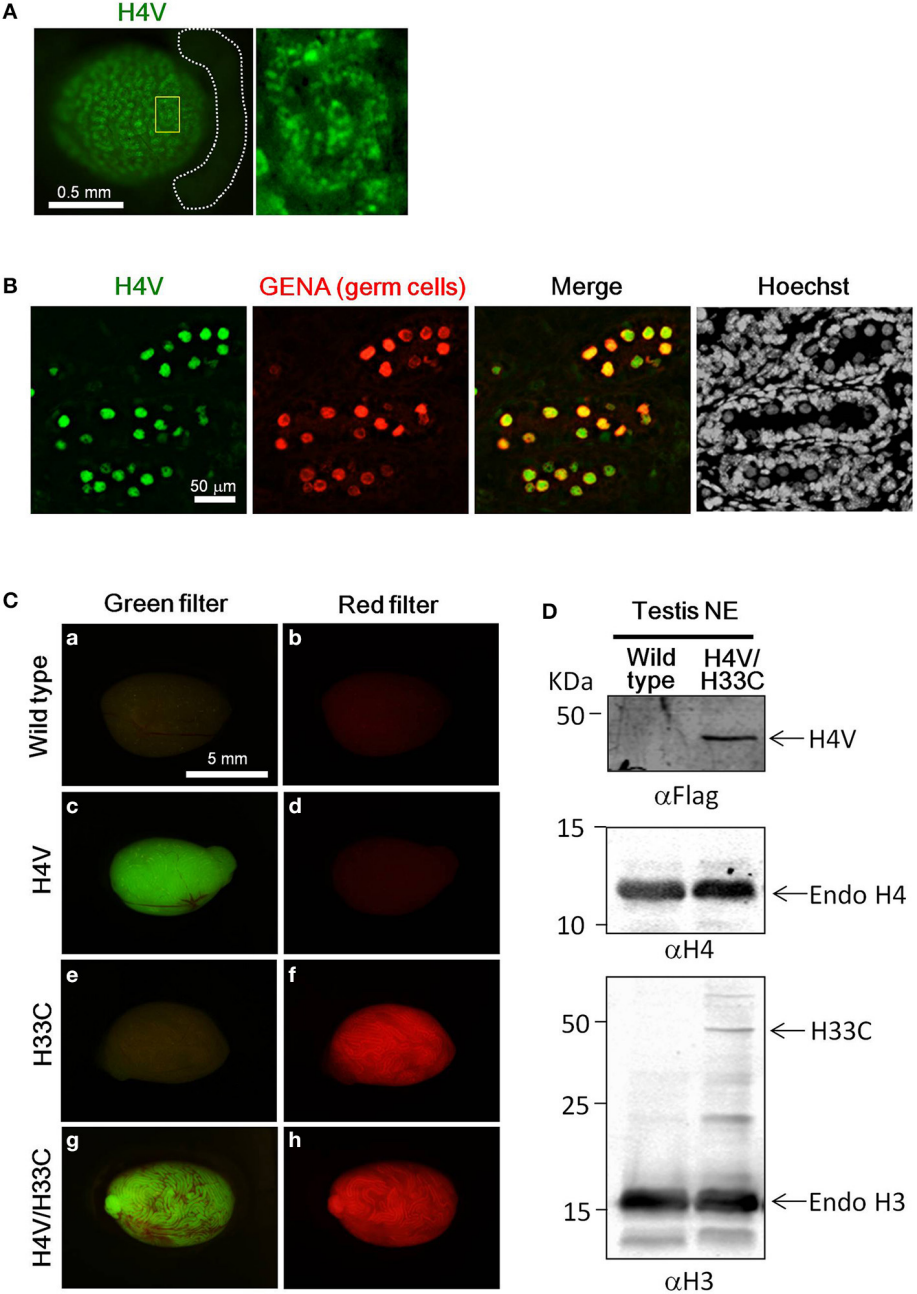
**Expression of H4V and H33C in mouse testes. (A)** Gross view of P0.5 testis of H4V Tg mouse (left panel). White dotted lines indicate the epididymis. Dotted distribution of H4V-positive cells is exhibited under higher magnification of an area surrounded by yellow line in the left panel (right panel). **(B)** Histological features of P0.5 H4V Tg testis. H4V expression was derived from the transgene (green), whereas GENA, a marker of germ cells (red), was visualized by immunostaining. **(C)** Gross view of adult testes of wild type **(a,b)**, H4V **(c,d)**, H33C **(e,f)**, and H4V/H33C double **(g,h)** Tg testes. Images of left panels were visualized using a green filter, and those of right panels were visualized using a red filter. Scale bars are as indicated. **(D)** Western blot analysis to confirm the expression of H4V and H33C in the double Tg testis. Primary antibodies used for each panel were indicated. Middle panel exhibiting the expression of endogenous H4 was served as a loading control of αFlag western blot (upper panel). NE, nuclear extract.

Both H4V and H33C Tg lines were healthy and fertile (data not shown). H4V signals were detectable in postnatal day (P) P0.5 neonatal testes as numerous dots in the seminiferous tubules (Figure 2A). These dotted pattern represented nuclear expression of H4V in gonocytes, since the perinatal germ cells which express GENA in their nuclei (Figure 2B). In the heterozygous adult testes, H4V and H33C signals, respectively, were strong enough to be detected macroscopically (Figures 2Ca–f). In the testes of double Tg mice obtained by crossing H4V and H33C, both transgenes were properly expressed (Figures 2Cg,h). In western blotting, the expression of H33C fusion protein was detected around the expected size (~44 KDa) using αH3 antibody (Figure 2D). On the other hand, H4V failed to be detected using αH4 antibody presumably due to its lower expression level (data not shown). Alternatively, H4V was detected around the expected size (~40 KDa) by using αFlag antibody (Figures 1A, 2D).

**Figure 3:**
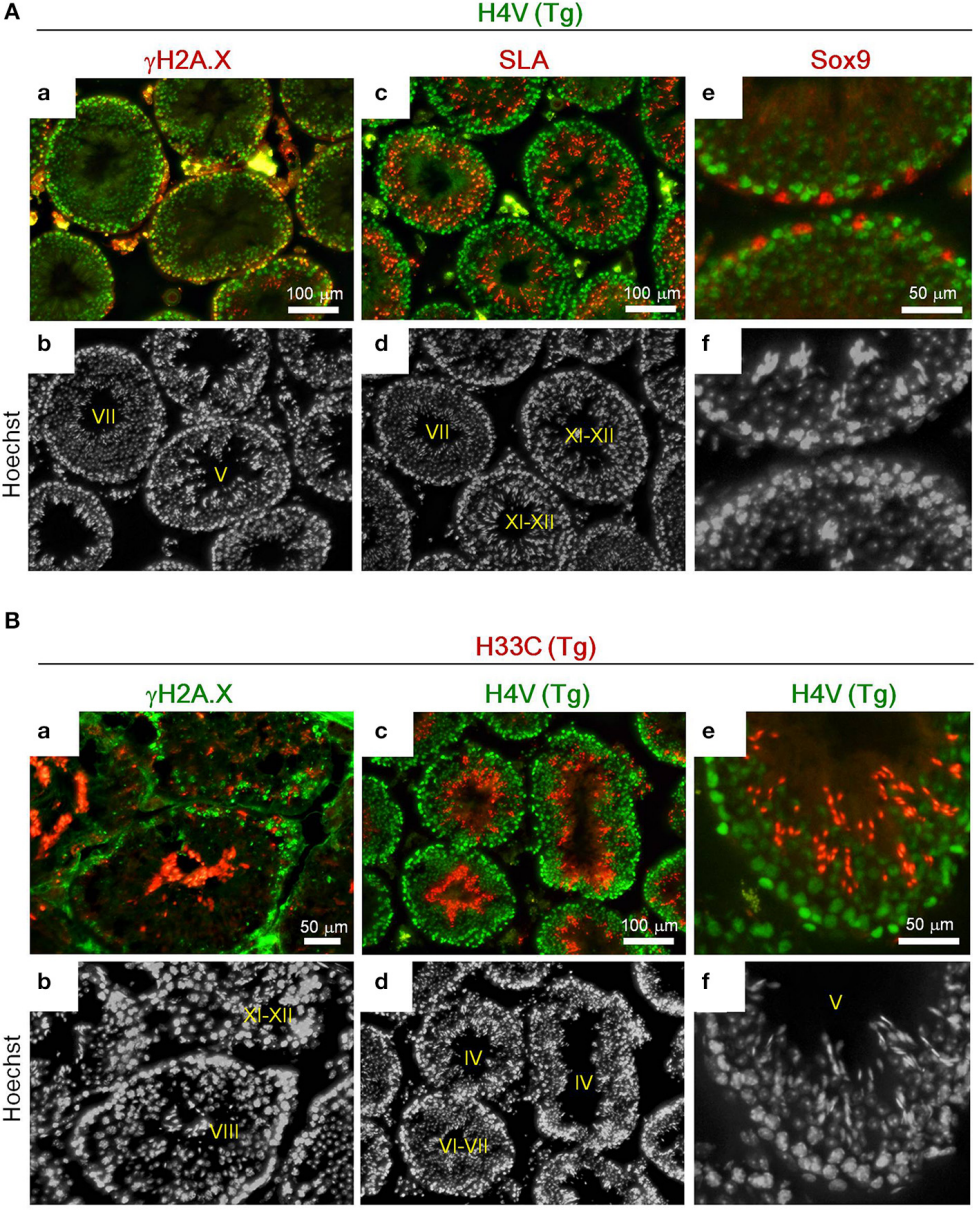
**Immunohistological analyses of the adult Tg testes. (A)** Expression of γH2A.X **(a,b)**, SLA **(c,d)**, and Sox9 **(e,f)** in testes of adult H4V Tg mice. Roman numerals indicate stages of spermatogenesis of the seminiferous tubules. **(B)** Expression of γH2A.X **(a,b)** and H4V **(c–f)** in testes of adult H33C Tg mice. H4V signals in **(c)** and **(e)** were derived from the transgene (i.e., the double Tg mice). Roman numerals indicate stages of spermatogenesis of the seminiferous tubules. Scale bars are as indicated.

Histologically, H4V was prominently expressed in the basal layer of seminiferous tubules including γH2A.X-positive spermatocytes (i.e., leptotene, zygotene, pachytene, and diplotene) (Figures 3Aa,b). Smaller cells attached to the basement membrane, which were likely spermatogonia, also possessed intense H4V (Figures 3Aa,b). Post-meiotic haploid spermatids marked by SLA also exhibited weaker H4V signals in certain stages in seminiferous tubules including Stage VII, but not in later stages such as Stage XI-XII (Figures 3Ac,d, 4C). Sertoli cells, the intra-tubular testicular somatic cells marked by Sox9, did not express H4V (Figures 3Ae,f). In contrast, expression of H33C existed mainly in the lumen side of seminiferous tubules. These cells were γH2A.X-negative, suggesting that these were post-meiotic spermatids (Figures 3Ba,b). Elongating spermatids in Stage XI–XII seminiferous tubules exhibited modest H33C signals compared with those of elongated spermatids in Stage VIII (Figures 3Ba,b), suggesting that the expression of H33C increased as the differentiation of spermatids proceeded. Interestingly, in H4V/H33C double Tg testes, germ cells with both signals were hardly observed in any spermatogenic stages (Figures 3Bc–f), implying mutually exclusive expression of H4V and H33C.

**Figure 4:**
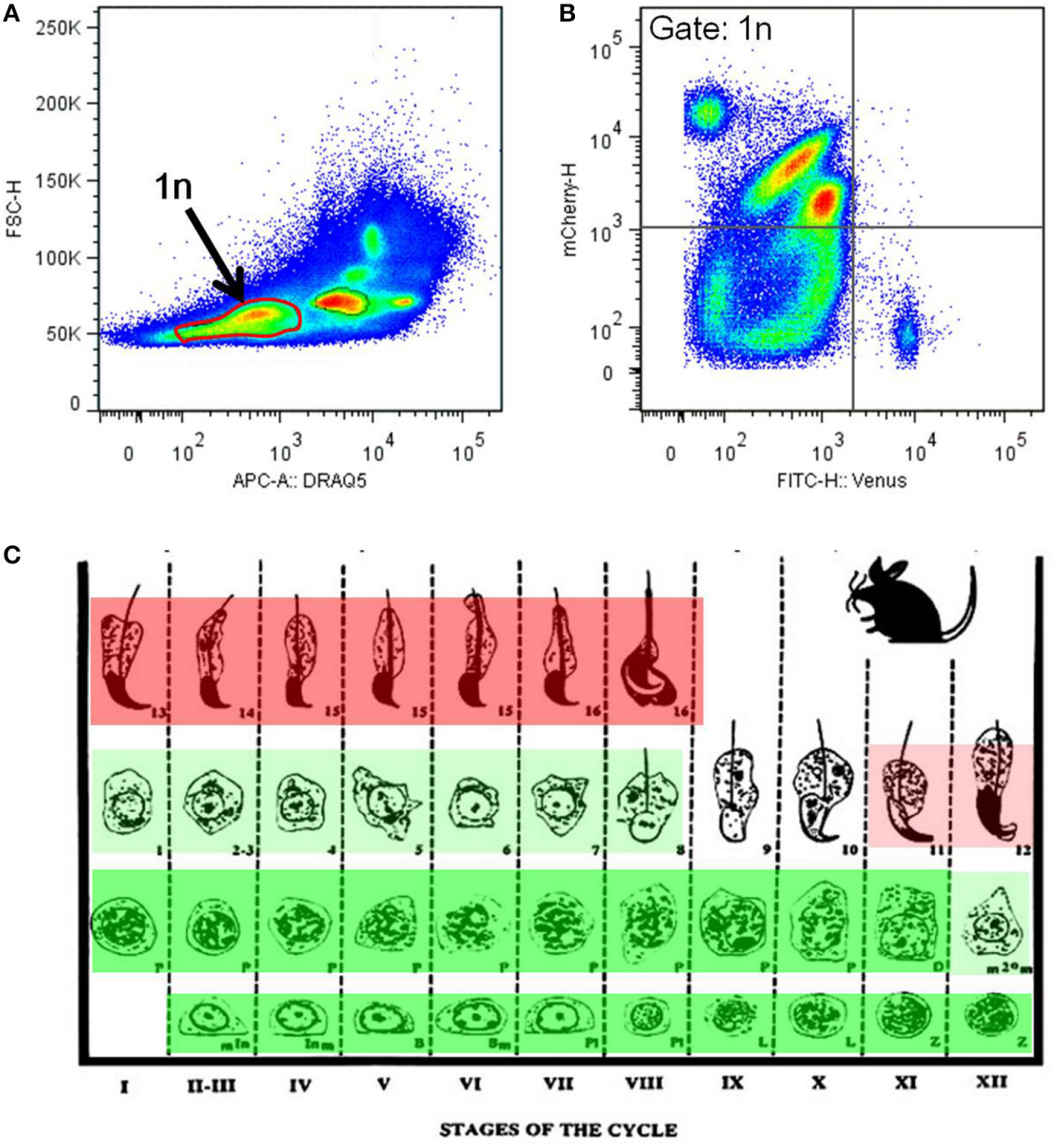
**Flow cytometry analysis of the H4V/H33C double Tg testes. (A)** Separation of testicular cells by the DNA contents. Approximately 30% of cells with low DRAQ5 fluorescence are assessed as post-meiotic haploid cells indicated as 1n. **(B)** Expression of Venus and mCherry in haploid cells gated in **(A)**. **(C)** Scheme of mouse spermatogenesis with expression patterns and the intensities of Venus (green) and mCherry (red). Roman numerals represent spermatogenic stages, and Arabic numerals indicate spermiogenic stages, respectively. This scheme is modified from Russell et al. (1990).

Flow cytometrical analysis was performed to confirm the mutual exclusivity between H4V and H33C. Post-meiotic haploid spermatids (1n) were isolated by DNA staining, and their H4V and H33C intensities were examined. We observed that the majority of haploid cells expressed only H33C, and consistent with this histological observation, H4V/H33C double-positive cells were hardly detected (Figures 4A,B). Taken together, these results suggest that H4V is intensely expressed in spermatogonia through approximately Step 8 of spermatids, whereas H33C starts to be detected around Step 11, and remains prominent until the final stage of testicular spermatogenesis (Figure 4C).

### Application of H4V/H33C double Tg cells for SSC transplantation assay

**Figure 5:**
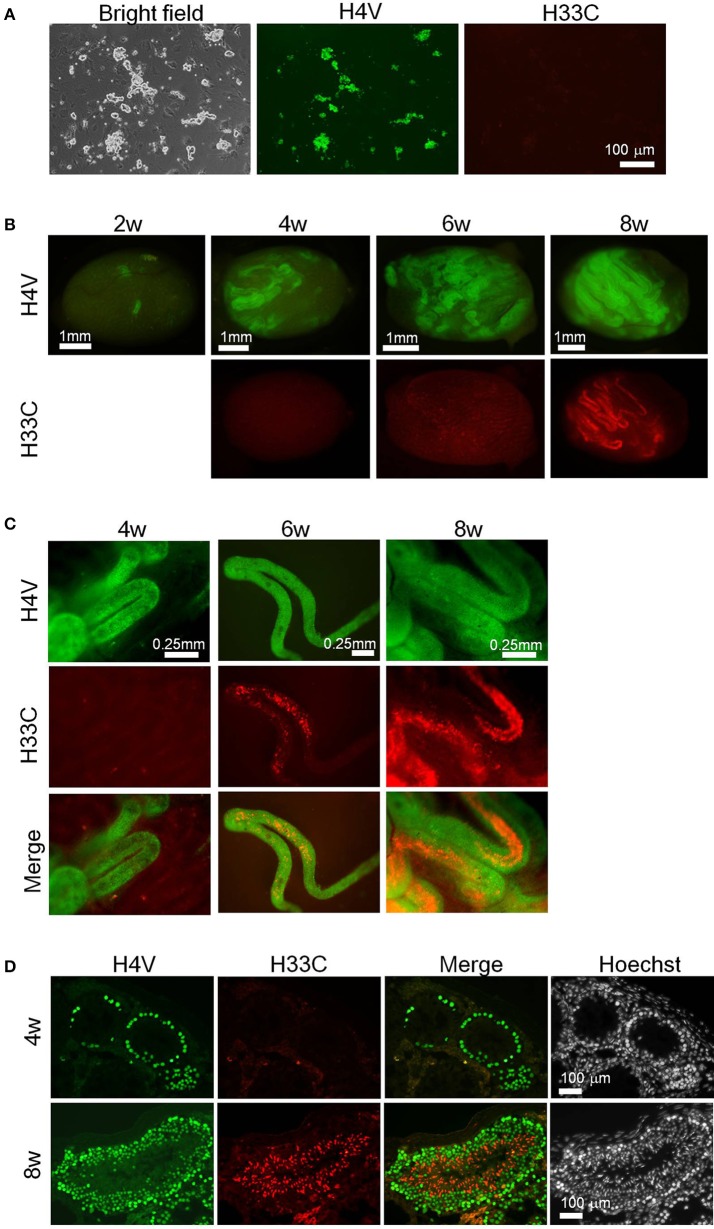
**Transplantation assay using H4V/H33C double Tg SSCs. (A)** Isolation of SSCs (= donor cells) from H4V/H33C double Tg mice. **(B)** Gross view of the recipient testes representing efficiency of the engraftment after transplantation. The examined timepoints post-transplantation are indicated. **(C)** Higher magnification of seminiferous tubules shown in **(B)**. **(D)** Histological features of the recipient testes at the indicated timepoints. Scale bars are as indicated.

Since the results above demonstrated an apparent switch of H4V and H33C during spermatogenesis, the double Tg cells seemed useful to monitor spermatogenesis *in vivo*. To test this possibility, we isolated SSCs from P9 pups of H4V/H33C double Tg mice, and subjected them to transplantation analysis. The SSCs cultured *in vitro* for 3–4 weeks exhibited “grape-shaped” colonies, which are typical for SSCs (Figure 5A). Consistent to the expression pattern in testes, the SSCs expressed only H4V, whereas H33C expression remained at background levels (Figure 5A). 1–2 × 10^5^ SSCs were transplanted to a testis of W/Wv mice, and the engrafted cells were examined every 2 weeks. At the second weeks post-transplantation, small clusters of H4V-positive cells were already detected in the recipient testes, indicating a successful engraftment (Figure 5B, see a panel of 2w). On the fourth week, H4V-positive clusters increased the number, but H33C expression had not been detected (Figures 5B,C, see 4w panels). On the sixth week, a small number of H33C-expressing cells started to form clusters in the seminiferous tubules (Figures 5B,C, see 6w panels). Finally at the eighth week, H33C-positive clusters were largely expanded (Figure 5B, see 8w panels), and exhibited a discontinuous cluster formation representing spermiogenic step-dependent expression (Figure 5C, see 8w panels). Consistently, histological examination revealed H4V-only expression at the fourth week, as engrafted cells had not entered the post-meiotic stages yet (Figure 5D, see the upper 4w panels), whereas at the eighth week, the engrafted cells proceeded to the later stages of post-meiosis, where H33C was predominantly expressed (Figure 5D, see the lower 8w panels). These results confirmed our hypothesis that the H4V/H33C double Tg cells were useful to monitor spermatogenesis *in vivo*.

**Figure 6:**
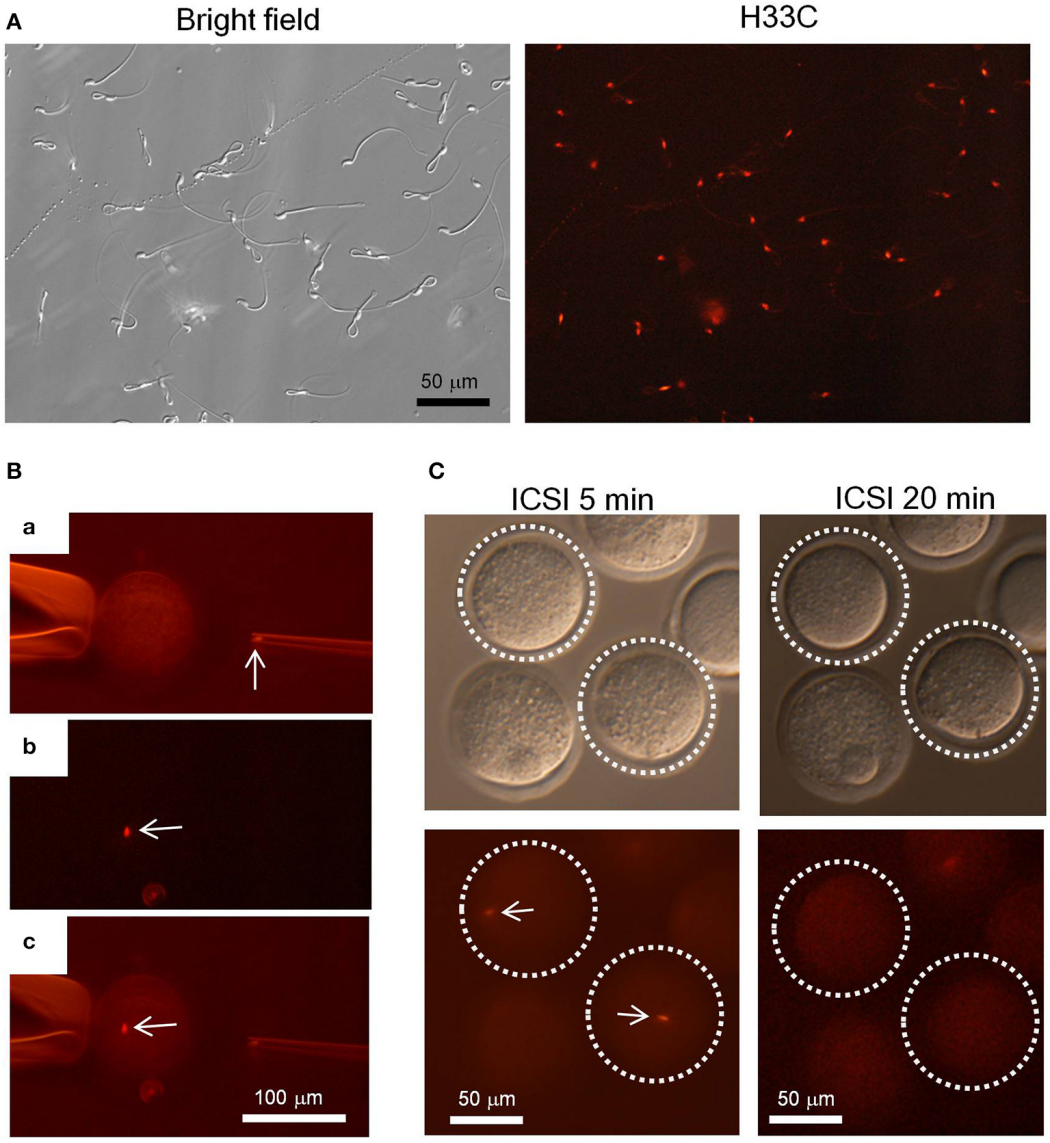
**Intra-cytoplasmic sperm injection (ICSI) using H33C sperm. (A)** Expression of H33C in the head of epididymal sperm. **(B)** A representative image of performing ICSI using H33C sperm. **(a)** An H33C-positive sperm head in an injection capillary before injection. **(b,c)** An H33C-positive sperm (white arrows) in ooplasm immediately after injection. **(C)** H33C-positive sperm-injected oocytes at the indicated timepoints. White dotted lines denote oocytes containing H33C-positive sperm (white arrows). Scale bars are as indicated.

Finally, we isolated epididymal sperm from H33C Tg mice to see whether H33C persists in spermatozoa, and found that almost all spermatozoa exhibited H33C prominently in their nuclei (Figure 6A). Interestingly, the fluorescence in sperm heads disappeared within 20 min, when they were injected into the cytoplasm of unfertilized oocytes (i.e., ICSI), indicating the dynamic remodeling of male chromatin after fertilization (Figures 6B,C).

## Discussion

In this study, we established H4V and H33C transgenic mouse lines, and demonstrated that their distinct expression patterns during spermatogenesis can be utilized for monitoring spermatogenesis *in vivo*. Recently, Sato et al developed an *in vitro* testicular organ culture system, which enables the real-time monitoring of spermatogenesis *in vitro* (Sato et al., 2011, 2013). Applying the testes of our Tg mice to this *in vitro* testicular organ culture system will allow us not only to determine the stages of spermatogenesis without further immunostaining, but also to perform real-time imaging of spermatogenesis. Particularly, as shown in Figure 5, using the double Tg SSCs for a transplantation assay is a convenient way to monitor engraftment efficiency, and to evaluate the differentiation stages of transplanted cells in real time.

In addition, these Tg lines can be a strong tool to study histone-protamine replacement during spermiogenesis. It is well known that in the nuclei of spermatids, histones are removed from chromatin en masse, and subjected to degradation in the course of spermiogenesis through their exchange with TNPs and protamines. Recently, Qian et al. demonstrated that a spermatid/sperm-specific proteasomal complex is responsible for histone degradation, and that acetylation of histones including H4 lysine 16 (H4K16) are required for binding histones to the proteasomal complex (Qian et al., 2013). On the other hand, it has also been reported that small amount of histones escape this removal, and are retained in spermatozoa (Hammoud et al., 2009). Because H3.3 is one of the histones selectively retained in spermatozoa (Erkek et al., 2013), our finding that H4V and H33C are mutually exclusive distributed in spermatids is likely to reflect the fine-tuned exchange of histone removal and deposition during spermiogenesis, although the relatively large size of the Venus tag for H4V, and the promoter choice for H33C expression may have had some artificial effects.

Similarly, we also observed a rapid removal of sperm H33C after fertilization, consistent with previous studies that reported that protamine withdrawal occurs 1–60 min after fertilization in different animal species due to protamine association to nucleoplasmin rather than their degradation (Ohsumi and Katagiri, 1991; Jones et al., 2011). In contrast, maternal-derived H3.3 is quickly incorporated into male pronuclei soon after fertilization (Torres-Padilla et al., 2006). Since the localization of H3.3 in the sperm genome was recently reported, it is a challenging but intrinsic task to determine the distribution of H3.3 in male pronuclei to see why the H3.3 needs to be exchanged by another H3.3 in such a short period of time.

Taken above together, we propose that our developed H4V and H33C Tg mice can serve as useful tools for further researches into spermatogenesis.

### Conflict of interest statement

The authors declare that the research was conducted in the absence of any commercial or financial relationships that could be construed as a potential conflict of interest.

## References

[B1] Aoshima K. Baba A. Makino Y. Okada Y. (2013). Establishment of alternative culture method for spermatogonial stem cells using knockout serum replacement. PLoS ONE 8:e77715. 10.1371/journal.pone.0077715 24204931 PMC3810131

[B2] Brinster R. L. Zimmermann J. W. (1994). Spermatogenesis following male germ-cell transplantation. Proc. Natl. Acad. Sci. U.S.A. 91, 11298–11302. 10.1073/pnas.91.24.11298 7972053 PMC45218

[B3] Chen C. H. Wang C. W. Hsu M. I. Huang Y. H. Lai W. F. Tzeng C. R. (2012). Bioluminescence imaging as a tool to evaluate germ cells *in vitro* and transplantation *in vivo* as fertility preservation of prepubertal male mice. Fertil. Steril. 97, 1192–1198. 10.1016/j.fertnstert.2012.02.003 22424616

[B4] Copeland N. G. Jenkins N. A. Court D. L. (2001). Recombineering: a powerful new tool for mouse functional genomics. Nat. Rev. Genet. 2, 769–779. 10.1038/35093556 11584293

[B5] Erkek S. Hisano M. Liang C. Y. Gill M. Murr R. Dieker J.. (2013). Molecular determinants of nucleosome retention at CpG-rich sequences in mouse spermatozoa. Nat. Struct. Mol. Biol. 20, 868–875. 10.1038/nsmb.2599 23770822

[B6] Hamer G. Roepers-Gajadien H. L. van Duyn-Goedhart A. Gademan I. S. Kal H. B. van Buul P. P.. (2003). DNA double-strand breaks and gamma-H2AX signaling in the testis. Biol. Reprod. 68, 628–634. 10.1095/biolreprod.102.008672 12533428

[B7] Hammoud S. S. Nix D. A. Zhang H. Purwar J. Carrell D. T. Cairns B. R. (2009). Distinctive chromatin in human sperm packages genes for embryo development. Nature 460, 473–478. 10.1038/nature08162 19525931 PMC2858064

[B8] Hara K. Nakagawa T. Enomoto H. Suzuki M. Yamamoto M. Simons B. D.. (2014). Mouse spermatogenic stem cells continually interconvert between equipotent singly isolated and syncytial states. Cell Stem Cell 14, 658–672. 10.1016/j.stem.2014.01.019 24792118 PMC4010676

[B9] Jones E. L. Zalensky A. O. Zalenskaya I. A. (2011). Protamine withdrawal from human sperm nuclei following heterologous ICSI into hamster oocytes. Protein Pept. Lett. 18, 811–816. 10.2174/092986611795713925 21443492

[B10] Nel-Themaat L. Jang C. W. Stewart M. D. Akiyama H. Viger R. S. Behringer R. R. (2011). Sertoli cell behaviors in developing testis cords and postnatal seminiferous tubules of the mouse. Biol. Reprod. 84, 342–350. 10.1095/biolreprod.110.086900 20944081 PMC3071268

[B11] Ohsumi K. Katagiri C. (1991). Characterization of the ooplasmic factor inducing decondensation of and protamine removal from toad sperm nuclei: involvement of nucleoplasmin. Dev. Biol. 148, 295–305. 10.1016/0012-1606(91)90338-4 1936566

[B12] Qian M. X. Pang Y. Liu C. H. Haratake K. Du B. Y. Ji D. Y.. (2013). Acetylation-mediated proteasomal degradation of core histones during DNA repair and spermatogenesis. Cell 153, 1012–1024. 10.1016/j.cell.2013.04.032 23706739 PMC3983474

[B13] Russell L. D. Ettlin R. A. Sinha Hikim A. P. Clegg E. D. (1990). Histological and Histopathological Evaluation of the Testis. St Louis, MO: Cache River Press

[B14] Sato T. Katagiri K. Gohbara A. Inoue K. Ogonuki N. Ogura A.. (2011). *In vitro* production of functional sperm in cultured neonatal mouse testes. Nature 471, 504–507. 10.1038/nature09850 21430778

[B15] Sato T. Katagiri K. Kubota Y. Ogawa T. (2013). *In vitro* sperm production from mouse spermatogonial stem cell lines using an organ culture method. Nat. Protoc. 8, 2098–2104. 10.1038/nprot.2013.138 24091557

[B16] Sumiyama K. Kawakami K. Yagita K. (2010). A simple and highly efficient transgenesis method in mice with the Tol2 transposon system and cytoplasmic microinjection. Genomics 95, 306–311. 10.1016/j.ygeno.2010.02.006 20219670

[B17] Torres-Padilla M. E. Bannister A. J. Hurd P. J. Kouzarides T. Zernicka-Goetz M. (2006). Dynamic distribution of the replacement histone variant H3.3 in the mouse oocyte and preimplantation embryos. Int. J. Dev. Biol. 50, 455–461. 10.1387/ijdb.052073mt 16586346

[B18] Yoshida S. (2012). Elucidating the identity and behavior of spermatogenic stem cells in the mouse testis. Reproduction 144, 293–302. 10.1530/REP-11-0320 22733803

[B19] Yusa K. Rad R. Takeda J. Bradley A. (2009). Generation of transgene-free induced pluripotent mouse stem cells by the piggyBac transposon. Nat. Methods 6, 363–369. 10.1038/nmeth.1323 19337237 PMC2677165

[B20] Yusa K. Zhou L. Li M. A. Bradley A. Craig N. L. (2011). A hyperactive piggyBac transposase for mammalian applications. Proc. Natl. Acad. Sci. U.S.A. 108, 1531–1536. 10.1073/pnas.1008322108 21205896 PMC3029773

